# From Biomedical Applications of Alginate towards CVD Implications Linked to COVID-19

**DOI:** 10.3390/ph15030318

**Published:** 2022-03-07

**Authors:** Angela Spoială, Cornelia-Ioana Ilie, Denisa Ficai, Anton Ficai, Ecaterina Andronescu

**Affiliations:** 1 Department of Science and Engineering of Oxide Materials and Nanomaterials, Faculty of Applied Chemistry and Materials Science, University Politehnica of Bucharest, 1-7 Gh Polizu Street, 011061 Bucharest, Romania; angela.spoiala@upb.ro (A.S.); cornelia_ioana.ilie@upb.ro (C.-I.I.); anton.ficai@upb.ro (A.F.); ecaterina.andronescu@upb.ro (E.A.); 2 National Centre for Micro and Nanomaterials and National Centre for Food Safety, Faculty of Applied Chemistry and Materials Science, University Politehnica of Bucharest, Spl. Independentei 313, 060042 Bucharest, Romania; 3 Department of Inorganic Chemistry, Physical Chemistry, and Electrochemistry, Faculty of Applied Chemistry and Materials Science, University Politehnica of Bucharest, 1-7 Gh Polizu Street, 050054 Bucharest, Romania; 4 Academy of Romanian Scientists, Ilfov Street 3, 050045 Bucharest, Romania

**Keywords:** alginate, biomedical applications, CVDs, COVID-19

## Abstract

In the past year, researchers have focused their attention on developing new strategies for understanding how the coronavirus affects human health and developing novel biomaterials to help patients with cardiovascular disease, which greatly increases the risk of complications from the virus. Natural biopolymers have been investigated, and it has been proven that alginate-based materials have important features. This review presents an overview of alginate-based materials used for developing innovative biomaterial platforms for biomedical applications to mitigate the effects of the coronavirus. As presented in this review, COVID-19 affects the cardiovascular system, not only the lungs. The first part of the review presents an introduction to cardiovascular diseases and describes how they have become an important problem worldwide. In the second part of the review, the origin and unique properties of the alginate biopolymer are presented. Among the properties of alginate, the most important are its biocompatibility, biodegradability, low cost, nontoxicity, unique structure, and interesting features after chemical modification. The third section of the review illustrates some of the functions of alginate in biomedical, pharmaceutical, and drug delivery applications. Researchers are using alginate to develop new devices and materials for repairing heart tissues that have been damaged by the coronavirus. Further, insights regarding how cardiovascular disease affects COVID-19 patients are also discussed. Finally, we conclude the review by presenting a summary of the impacts of COVID-19 on cardiovascular patients, their implications, and several hypothetical alginate-based treatments for infected patients.

## 1. Introduction

Cardiovascular diseases (CVDs) are the leading cause of death worldwide. According to the World Health Organization, more than 17.9 million people died from CVDs in 2016, which represents almost 30% of all global deaths, and 85% of these deaths were caused by heart attacks and strokes. CVDs are conditions of the heart and bloodstream and include coronary heart disease, cerebrovascular disease, rheumatic heart disease, deep vein thrombosis, pulmonary embolism, and other diseases. Someone with a high risk of CVDs may exhibit elevated blood pressure, high glucose, and overweight or obesity; the next step in preventing a heart attack or a stroke must be receiving appropriate treatment, which could substantially contribute to preventing premature death [1].

As a rising problem that is affecting an increasing proportion of the population, CVDs have a great impact in low- and middle-income countries; worldwide health organizations need to develop effective prevention programs to monitor the importance of risk factors in diverse geographic areas and various ethnic groups. An international, standardized control study named INTERHEART was designed to prevent cardiovascular risks by evaluating the importance of risk factors for coronary disease worldwide. About 15,000 people from 52 countries participated in the study, whose main objective was to determine associations between various risk factors and to prevent acute myocardial infarctions in the population that participated in the study [2].

The outbreak of coronavirus disease 2019 (COVID-19), a serious infectious disease caused by severe acute respiratory disease coronavirus 2 (SARS-CoV-2), has affected human health worldwide. The novel coronavirus of debatable origin rapidly spread throughout the world, and to date, 426,747,804 cases and 5,911,499 deaths have been reported, according to Worldometer [3]. Although public health sectors implemented several control strategies, such as social distancing, hygienic measures, and the development of antiviral drugs and vaccines, the pandemic is still a critical challenge [4]. China’s Centre for Disease Control and Prevention has reported that CVDs increase the incidence and severity of coronavirus infection. Previous studies suggested that the presence of different heart diseases was an important risk factor for severe coronavirus infection in young children. Several case studies of COVID-19 patients revealed cardiac complications such as electrocardiography abnormities and diastolic dysfunction and suggested that the most commonly damaged organ, other than the lungs, was the heart. Therefore, it is extremely important to pay more attention to the cardiovascular effects of COVID-19 and the further development of specific treatments [3,5].

Given the high incidence of CVDs, researchers have been attempting to develop novel biomaterials to overcome cardiac complications that damage the heart. As a result, it was found that alginate has numerous applications for heart and cardiovascular diseases. Alginate biopolymer has been extensively researched for many biomedical applications due to its biocompatibility, low toxicity, cost-effectiveness, biodegradability, and nontoxicity. The Food and Drug Administration (FDA) approved alginate for multiple and versatile biomedical applications. Alginate hydrogels present beneficial properties in cardiovascular applications due to their low reactivity in vivo, low cost, non-thrombogenic nature, mild gelation conditions, and similarity to extracellular matrices [5]. In their work, Xu et al. [6] presented the latest views on alginate-based applications in the treatment of cardiovascular disease. Two injectable alginate implants have already reached clinical trials, showing the promising potential of alginate in myocardial repair and regeneration [7,8,9,10].

It is worth mentioning that the first study was conducted on alginate containing reduced graphene oxide for cardiac repair [11]. The purpose of this study was to benefit from the specific properties of alginate. The researchers showed that the exceptional mechanical and biological properties of alginate could improve cell-material interaction and provide a suitable platform for myocardial infarction treatment. The study also revealed that reduced graphene oxide contributed to the good biocompatibility of alginate for developing new platforms for cardiac repair [11].

The main aim of this manuscript is to highlight the potential use of alginate-based solutions in the treatment of CVDs, especially those associated with COVID-19. The review is based on the extensive literature on CVDs, as well as the recent literature on COVID-19 and its association with CVDs.

## 2. Alginate: Origin and Properties

**Figure 1:**
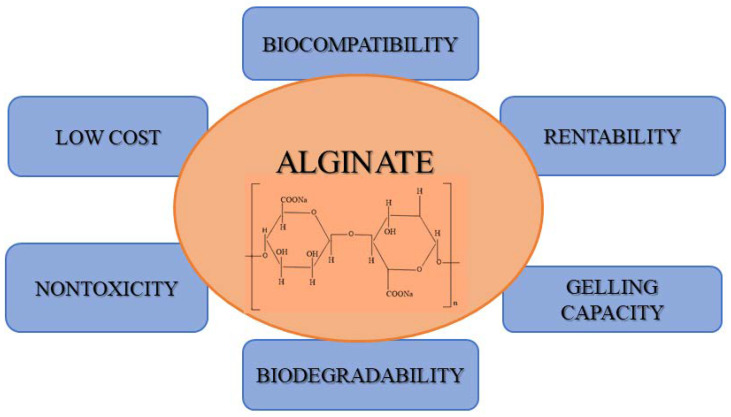
Alginate’s properties and its structure.

Alginate is a natural anionic biopolymer originating in brown algae and, due to its properties, has become an important biomaterial with interesting applications. Some of its properties, such as biocompatibility, biodegradability, low production cost, low toxicity, and gelation properties, along with antimicrobial and anti-inflammatory activity, suggest that alginate is a promising material for many applications in diverse fields of biotechnology, including the biomedical, medical, and food and beverage industries. Figure 1 illustrates the interconnection of alginate’s properties and its structure. In addition to the mentioned properties, alginate has other unique characteristics that make it useful as a matrix for delivery systems or the entrapment of diverse biomolecules [12,13,14,15,16].

Alginate is derived from two microbial sources: algae and bacteria [17]. It is well known that alginate is secreted by two genera of bacteria, which are *Pseudomonas* and *Azotobacter*, but the polysaccharide-based alginate is found in *Pseudomonas aeruginosa* [18,19]. Additionally, these two types of bacteria differ in the biosynthesis of alginate, and each produces alginate with different properties and different purposes. *P. aeruginosa* mucoid strains can produce high quantities of alginate used in the production of biofilms [20,21], while *Azotobacter* produces harder alginate due to the higher concentration of guluronate in the alginate structure, which permits the growth of resistant cells [22]. It has been discovered that alginate can also be synthesized from a bacterial strain named *A. vinelandii* through an encystment process, which is a process that allows bacteria to survive under harsh conditions. Alginate is also produced by another *Azotobacter* species, *Azotobacter chroococcum*, but currently, biotechnological applications require alginate isolated from algal sources [23].

This section presents the physical and chemical properties of alginate that make it useful in many food and industrial applications. Among these features are its ability to retain water and easily form gels, viscosity, stability emulsification, and sol–gel transition in the presence of cations, such as divalent cations, including calcium [24]. The chemical composition and physical properties of alginate vary depending on factors such as species, structure, environmental conditions, and even color, which means that its most suitable form can be selected for a given application [25]. Alginate salts are either white or yellowish-brown powders, which are odorless and flavorless. Alginates are copolymer blocks composed of two uronic acids, mannuronic acid (M-block) and guluronic acid (G-block), with different compositions and arrangements. It is known that there is a correlation between the arrangement of the uronic acid blocks, the age of the source organism, and the growth conditions. For instance, the algae *Laminaria hyperborea* contains a high quantity of guluronic acid when it grows away from coastal areas, whereas the same species floating in water has lower guluronic content [26].

When it comes to the chemical properties, alginate’s solubility is influenced by factors such as calcium concentration and pH. The ability of alginate to form gels is attributed to intermolecular interactions between calcium cations and polyguluronate sites of the alginate molecule. Cross-linking between carboxyl groups and divalent cations is the foundation of gel formation. Therefore, the sol-gel transition of alginate is not temperature-dependent, and alginate gels can be heated without melting. The properties of alginate solutions can also be tailored according to the application. One of these properties is concentration, which can have a high viscosity of 2000 cps at 1% concentration in water or a very low viscosity of less than 10 cps at the same concentration. The hydrophilicity, nontoxicity, biocompatibility, low cost, and water solubility have attracted researchers’ attention in recent years. The reversible solubility of alginate enables its synthesis in different forms, such as fibers, films, and microspheres, and the low mechanical strength of alginate has been improved by blending it with other nontoxic biopolymers. It is worth mentioning that due to their special properties and chemistry, alginate gels have become important biopolymers in different biomedical applications, as presented in the next section of the review [13].

## 3. Biomedical Applications of Alginate

**Figure 2:**
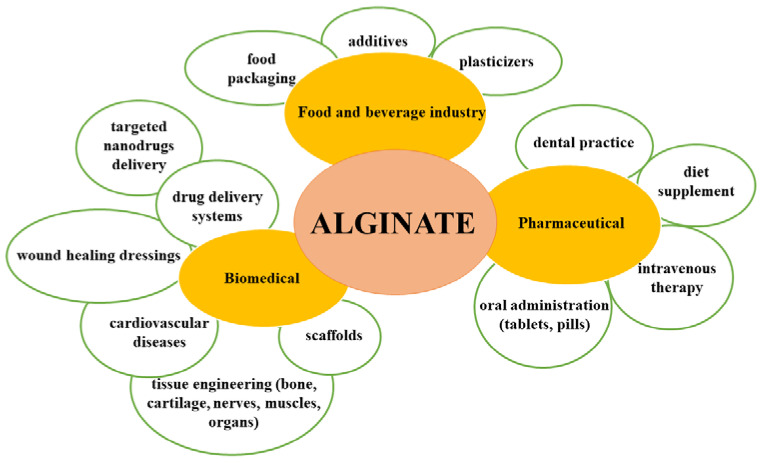
Highlighted biomedical applications of alginate.

When it comes to the applications of alginate (Figure 2), its natural anionic nature is responsible for many properties that make it attractive for usage in diverse industries. Alginates have several advantageous characteristics: they are low in cost, have high biocompatibility and low toxicity, and importantly, easily form gels. Moreover, the carboxyl and hydroxyl groups of alginate facilitate chemical and biochemical modifications that improve its properties and even reduce some of its disadvantages [27].

This section presents some of the biomedical applications of alginate, including in the pharmaceutical industry and even dental practice. Due to its properties, alginate is used as a thickener, stabilizer, or gel-forming agent in the pharmaceutical industry, as well as a material for components in dentistry and targeted drug delivery to diverse tissues. To control the release of drugs at a targeted site, the most important aspect is to point the drugs in the desired direction by selecting a suitable cross-linker agent and bonding method. There are different possible administration routes of alginate hydrogels loaded with drugs, including oral administration and local injections, allowing its use in many applications in the pharmaceutical industry [19,28].

In pharmaceutical applications, alginate is most frequently used for oral preparations, as capsules and tablets containing alginate are designed for the immediate or controlled release of drugs. Alginate is used in the tablet formulation because it protects the ingested drug from gastric acid [29]. The alginate matrix was investigated as a drug delivery vehicle for small molecule drugs or large biomolecules such as proteins and genes [30,31]. Many studies have confirmed that using alginate for protein drug delivery has advantages, such as mild conditions for protein incorporation and protection from degradation; the most important advantage is associated with its mucoadhesive capacity, which increases the bioavailability of the encapsulated protein and a decrease in the spread of the disease [19,32]. The use of alginate for the release of proteins and drugs also has some drawbacks, such as the rapid release of many proteins and the instability of the alginate matrix at high pH, which can cause rapid dissolution and the immediate release of active agents. To resolve these drawbacks, researchers found that the release rate could be tuned by increasing the gel porosity, drying the alginate beads, or using an appropriate cross-linking agent [19,33]. It is well known that alginates are extensively used for the encapsulation of a wide range of biologically active agents, such as melatonin [34], heparin [35], hemoglobin [36], vaccines [37], doxorubicin [38], lysozyme [39], adenoviruses [40], and most of all, insulin [41].

Additional examples of biomedical applications of alginate, such as wound healing treatment and bone, cartilage, cardiovascular, nerve, and tissue engineering applications, are presented next.

Alginate’s unique properties make it promising for many biomedical applications [19]. To be used in biomedical applications, alginate has to fulfil specific requirements, such as mechanical and chemical stability, narrow pore size, controllable swelling and degradability, and nontoxicity [42].

Tissue engineering involves the use of a natural or artificial substitute to assist in the regeneration, replacement, or repair of tissue or organ damage caused by injury or disease. Usually, repair and replacement of the damaged tissue or organ are used when surgery and implants succeed, but even then, particular materials that can replace or regenerate that tissue must be developed [43]. Scaffolds are extensively used in tissue engineering, the goal of which is to regenerate or repair organs or tissues in patients through cell delivery. For this purpose, hydrogels such as alginate are ideal choices; alginate can ensure sufficient space for tissue development and control the structure and functions of the formed tissue. Therefore, a profound understanding of the biology of the tissue and its extracellular matrix is necessary to be able to realize effective tissue regeneration [10]. For effective replacement, the scaffold should have specific attributes, such as physical strength to support cells, nontoxicity, non-immunogenicity, and suitable degradation; in other words, it needs to be biomimetic [44].

Alginate in gel form, alone and in combination with other polymers, was successfully tested as a biomaterial scaffold to carry and deliver proteins or cells that can help in the regeneration and repair of the damaged organs or tissues [19,45]. Alginate is one of the most promising and studied biomaterials for tissue engineering for many reasons, such as its biodegradability, biocompatibility, ability to be easily shaped low inflammatory response, and elimination through the kidneys. However, alginate functionalization is still very important when it comes to tissue engineering applications, so its functionalization through chemical modification is critical for the improvement of cell adhesion [46]. Despite all of its beneficial properties, alginate has its drawbacks, such as its low mechanical strength, which is not sufficient to support the structure of the regenerated tissue. The most suitable approach to solve these difficulties is to combine it with other biomaterials to improve its mechanical properties [47]. Notably, one important technique that has potential application in tissue engineering is inkjet printing, which uses synthetic 3D biodegradable hydrogel scaffolds for the reconstruction process. Alginate gels are predominantly used for cell delivery to regenerate or repair the bone, cartilage, blood vessels, organs, and other tissues [48,49].

**Figure 3:**
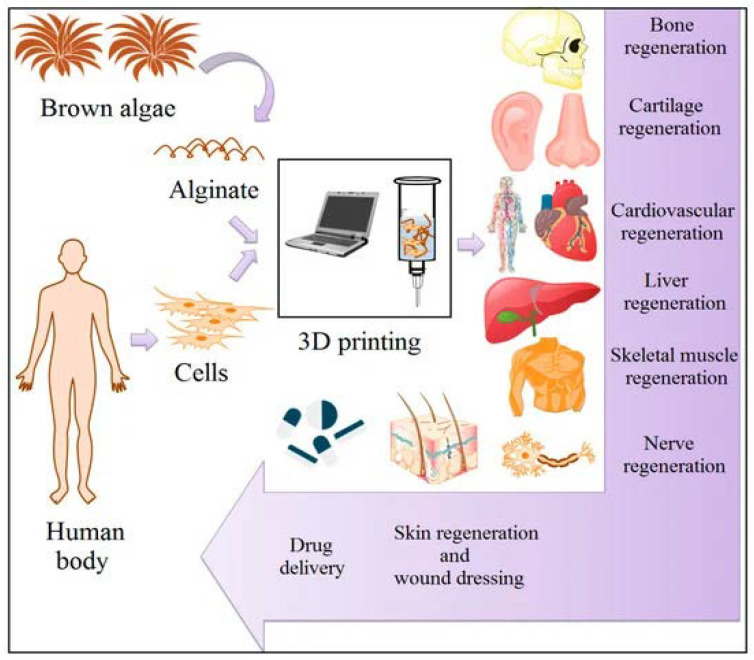
Three-dimensional alginate hydrogels in tissue engineering applications [60].

Alginate also has applications in repairing bone defects and supplying temporary scaffolds to treat these defects. These scaffolds are implanted with cells or designed to induce bone formation. For this purpose, certain bioactive substances, such as growth factors, proteins, drugs, or other agents that accelerate bone regeneration formation, are loaded and released [50]. Further, bone tissue engineering studies have examined different alginate scaffolds [51] and composites of alginate with diverse polymers [52]; proteins, including collagen and gelatine [53,54]; silica [55]; bioglass [56]; and peptides [57]. To use alginate biomaterials in scaffolds for cartilage tissue engineering, it must meet requirements such as mechanical stability and degradation rate. One of the best approaches remains the 3D bioprinting technique, which can overcome the issue of mechanical stability. Based on current knowledge and technology, this cannot be accomplished without combining different materials with alginate to produce the appropriate gel and using injectable hydrogels for in situ 3D printing [58,59]. Nevertheless, the composition is a key factor, and the control of the morpho-structural characteristics is currently exploited to improve the final performance. For this reason, additive manufacturing processes, especially 3D printing, are used. The 3D printing approach is considered an innovative technique for alginate’s revolutionary manufacturing in designing biomaterial platforms for tissue engineering applications to create the proper microstructure and finally to ensure the adequate release profile of biological active agents, the resorption of the support, cell internalization, etc. Some of the potential applications of alginate-based materials presented in the table below are also related to the applications presented in Figure 3 [60].

One focus of this review is the application of alginate biopolymer in cardiovascular tissue. The regeneration and replacement of cardiovascular tissue are among the most challenging approaches in the field of tissue engineering. Like many other applications of alginate, using alginate hydrogels with the 3D bioprinting technique appears to be promising for the regeneration of cardiac and vascular tissues [61]. Alginate hydrogels have also been used for engineering many other tissues and organs, such as the liver [62], muscle [63], skin [64], and adipose tissue [65]. Table 1 presents some of the potential applications of alginate-based materials.

## 4. Drug Delivery Applications of Alginate

Alginate is a naturally occurring polysaccharide obtained from brown algae or bacterial strains. Lately, alginate has been used more extensively as a matrix system for drug delivery or cell entrapment applications. As mentioned before, alginate is biodegradable, can be processed to achieve high porosity, and is highly mucoadhesive, which is why it is extensively used in drug delivery, including in formulations for oral administration [12]. For drug delivery applications, alginate has been used in combination with other materials, including other biopolymers or even inorganic materials such as clays, and these formulations have been proposed to induce efficient drug entrapment and release [70]. An appropriate combination can assure targeted delivery of bioactive agents, as, for instance, is presented by Iliescu et al. [70,75], who studied montmorillonite loaded with chemotherapeutic agents entrapped in an alginate matrix to ensure targeted delivery in the intestinal fluids and not in the stomach.

The anionic nature of alginate, in addition to its viscosity, defines its ability to attract cations and form gels [67]. To be used in diverse industries such as biomedicine, pharmaceuticals, and tissue engineering, alginate is often chemically modified to adjust its solubility and tailor its properties to each application [76]. Recent studies revealed that van der Waals forces within the alginate network lead to a three-dimensional (3D) gel structure of alginate [77].

The hydroxyl- and carboxylate-rich structure of alginate allows strong interactions (hydrogen bonds and ionic bonds) with drugs, and this has an important impact on the kinetics of drug release. Therefore, alginate gels have been considered a significant carrier for the controlled delivery of small molecule drugs by enhancing the diffusion of the molecules through the hydrogel [19]. Alginate has been used as a hydrophilic matrix in oral drug delivery. The transformation of sodium alginate into alginic acid and the porosity of the alginate matrix affect the drug release mechanism [71,78]. Alginate is also used as an adjuvant in antiacid formulations in treatments of gastric reflux, reducing gastroesophageal reflux by acting as a barrier. Due to the safety and long period of release, it can be used in infants and even during pregnancy to treat heartburn with no side effects, such as the anti-reflux formulation Gaviscon [79].

In addition, nanoparticles of doxorubicin-loaded glycyrrhetinic acid modified with alginate were developed to target only the cancerous liver tissue in cancer therapy. It has been reported that free doxorubicin has a high risk of cardiovascular side effects. The results show that alginate-based formulations have a reduced cardiovascular impact compared to free doxorubicin [72]. Alginate was considered the perfect candidate for the delivery of drugs as well as proteins. The encapsulation of proteins can preserve their 3D structure and protect them from degradation in an external biological environment. For example, different strategies for controlling protein release from alginate gels, such as the delivery of DNA, enzymes, and even hormones, were studied [12].

Alginate is used in wound dressings and has proved to be superior to other dressings when applied in the treatment of diabetic foot lesions. Wound healing can be a particular challenge in diabetic patients, and to overcome this problem, alginate wound dressings have been developed for both the prevention and healing of the treatment of foot lesions [73]. It has also been observed that alginate alone has a positive effect on wound healing, and it has proved to be an effective delivery system as well as a scaffold for wound healing treatment [74].

To conclude, alginate has been studied in various fields, including pharmaceuticals and biomedical devices. Recent studies have revealed that alginate applications have considerably increased, especially in scaffold development. Due to its impressive features, such as biocompatibility, biodegradability, nontoxicity, easy gelation, and chemical modification, alginate has been widely used in biomedical and pharmaceutical applications. Alginate has also been reported to be an exceptional vehicle for diverse biomolecules, such as DNA, proteins, and cells. The next section of the review introduces various examples of alginate applications in the design of drug delivery nanovehicles [12,70].

## 5. Applications of Alginate in CVDs

The use of alginate has also shifted toward cardiovascular applications. This section of the review covers the biomedical application of alginate in designing new platforms for CVDs, presenting the new devices and materials created for repairing damaged heart tissues [6].

Alginate hydrogels were used in treatments of myocardial infarction while investigating the inflammatory response, which occurs with the enlargement of the ventricular chamber and heart wall thinning. As a result, heart functions fail, and cardiac performance declines [80]. Alginate’s biodegradability was explored to evaluate its potential role in treating damaged myocardial tissue [66]. Zhang et al. [81] demonstrated that alginate-based hydrogels are a promising extracellular matrix substitute in transplanted cardiomyocytes in rats due to similarities of alginate to the extracellular matrix of the developing heart. Further investigation on the subject was conducted by applying alginate for myocardial tissue regeneration, and the results were promising. Alginate-based hydrogels were designed to mimic cardiac extracellular matrix properties according to their chemical, mechanical, and morphological characteristics. Due to alginate’s unique properties, it can offer short-term support until the heart heals and, most importantly, facilitate myocardial repair [82]. Recent studies confirmed the efficacy of alginate biomaterial injection into myocardial tissue, supporting regeneration and preserving cardiac function [83].

As a result of its extraordinary physical properties, alginate hydrogel substantially improved cardiac functions, showing promise as an artificial biomimetic material for cardiac extracellular matrix [84]. Levit et al. [85] encapsulated human bone marrow mesenchymal stem cells in alginate hydrogels to deliver them to damaged rat hearts in a biocompatible poly (ethylene glycol) (PEG) hydrogel patch to guarantee the safety of the cells. After multiple analyses, the results showed significantly improved cardiac functions and scar size reduction.

Alginate scaffolds with large pore sizes and high matrix porosity can accept a high load on their pores, which makes them an ideal 3D solid platform for in vitro cardiac tissue. Bioengineering and fabricating such 3D tissue grafts could perfectly recreate the 3D environment of the damaged tissue, enhancing vascularization and facilitating the organization of the tissue structure [86,87].

Another reported example of alginate hydrogels is the development of 3D nanocomposites of gold nanowires inside microporous alginate scaffolds, the purpose of which is to engineer bridges between the interconnected pore walls to increase electrical signalling through the entire scaffold and increase cardiac tissue functions [88].

Nagar et al. combined the benefits of reduced graphene oxide and alginate hydrogels for developing a promising hydrogel biomaterial for cardiac tissue engineering applications. The obtained results show that graphene oxide incorporated into alginate hydrogels enhanced gel stiffness, and the cytocompatibility of hydrogels prepared with human bone marrow-derived mesenchymal stem cells was confirmed. The tests also confirmed that the presence of reduced graphene oxide in alginate facilitated the development of a suitable hydrogel for stem cell therapy in heart disease patients [11].

Innovative three-layered alginate–graphene oxide/PCL scaffold was manufactured for the first time through the electrospinning method for potential use in cardiac tissue engineering. The top and bottom of the biomaterial were composed of alginate–graphene oxide (GO) nanofibrous layers for attachment and cell viability, and the middle was composed of a PCL layer that provided mechanical support to the scaffold. This study aimed to fabricate a nanofibrous scaffold to simulate the cardiac extracellular matrix (ECM). The overall results confirmed that the incorporation of GO into the alginate nanofibers enhanced cell adhesion, viability, and proliferation. In conclusion, the three-layered nanofibrous scaffold made of alginate, graphene oxide, and PCL could be considered a promising biomaterial for cardiac tissue regeneration [89].

Mesenchymal stem cells (MSCs) have been recognized as an attractive resource for the regeneration of infarcted myocardium, especially for their ability to synthesize cardiac-regenerating cytokines [90,91]. However, because of the inefficiency of cell-based therapy, mostly due to the low viability and poor therapeutic activity of the MSCs, researchers have improved their survival and therapeutic activity through implantation/encapsulation in hydrogels. The encapsulation technique offers multiple advantages enabling better protection for stem cell delivery, and it has been proven that cells inserted in hydrophilic and biocompatible polymer hydrogels are the best solution. This study reported that the novel encapsulation of MSCs in graphene oxide–alginate composite hydrogels exhibited cell protection effects (exhibiting antioxidant activity and protecting the cells from oxidative stress), which will serve as an efficient platform for the delivery of stem cells and the treatment of diverse diseases, including myocardial infarction [92].

Biomaterials such as alginate hydrogels can be designed to produce solid delivery systems for bioactive molecules such as growth factors, cytokines, and stem cells. To develop systems to encapsulate these types of bioactive molecules, biomaterials could serve as temporary vehicles that induce the formation and regeneration of cardiac tissues [6].

As we have seen already, alginate has shown its great utility and potential advantages as a biomaterial for use in cardiovascular disease, especially in the applications of cardiac tissue formation, stem cell delivery, encapsulation, and controlled release of bioactive molecules and 3D scaffolds. Alginate has produced successes that yielded great accomplishments in the field of cardiovascular applications, indicating promising developments toward achieving the perfect synergism between tissue reparation and regeneration [6,27].

## 6. COVID-19 Consequences in CVD Patients

It has been discovered that coronavirus disease affects the cardiovascular system in addition to the respiratory system. Studies in COVID-19 patients have led to the recognition of several stages in the disease course. The first stage is the appearance of major symptoms of viral infection, such as fever and cough. The second stage is characterized by respiratory failure and acute respiratory distress syndrome. The third and final stage, which worries cardiologists, affects the cardiovascular system. Researchers have concluded that cardiovascular risks are frequent among patients affected by COVID-19. Additionally, it has been found that heart failure and cardiogenic shock are potential effects of coronavirus disease, and reports involving monitoring and treating patients are still being investigated [93].

Recent discoveries involving coronavirus disease have led to the urgent need to develop new methods to prevent and treat COVID-19. Among the important research fields involved are biomaterial science and engineering. Biomaterials provide numerous opportunities to develop new modalities to prevent, diagnose, treat, and monitor diseases, as well as design vaccines. The most widespread infections are those caused by viruses. Thus, biomaterials have been used for developing antiviral platforms for the diagnosis and management of COVID-19 [94]. Biomaterials have been exploited for the treatment and diagnosis of COVID-19. To provide a suitable platform model to analyze the virus, it is very important to develop new biomaterials with antiviral properties. Tissue-engineered platforms can be excellent devices to design 3D bio-platforms to treat COVID-19. Using the 3D bioprinting method, bioink hydrogel-based alginate scaffolds were investigated in studies of cell responses to viral infection. It was demonstrated that hydrogel-based alginates were useful tools to investigate, develop, and identify novel drugs against SARS-CoV-2 infection [95,96].

Many studies have proved that alginate-based materials have antiviral activity against a wide range of viruses, such as double-stranded DNA viruses, positive-sense or negative-sense single-stranded RNA viruses, and single-stranded RNA viruses with DNA as an intermediate. These viruses can infect diverse organisms and include enveloped viruses in Baltimore group IV, to which SARS-CoV-2 belongs. Alginate-based materials show great promise for the treatment of COVID-19 disease. Alginate-based compounds demonstrated antiviral activity against the enveloped Baltimore group IV rubella virus (RV) infection in Vero cells. The results suggested that alginate’s inhibitory effect on RV was dose-dependent [97]. Alginate hydrogels have also shown an antiviral capacity against herpes simplex virus type 1 (HSV-1), and in vitro assessments indicated that sulfated alginate is a promising candidate for further antiviral research [98]. In addition, alginate-based materials were tested on diverse types of viruses, and they were found to have little or no cytotoxic effect. The antiviral mechanism of action is mainly attributed to viral aggregation and viral inhibition through the interconnection of alginate-based materials with components of the viral load [99]. Therefore, these studies suggest great opportunities for future research in the field of alginate-based biomaterials with antiviral properties against SARS-CoV-2 [96,100,101].

Driven by the urgent need to find a cure or to ease the effects of the pandemic on ill patients, researchers have proven that alginate acts as an immune activator in diverse in vivo animal models and in vitro cell systems, which could lead to important findings for the therapeutic management of COVID-19 patients. The researchers of this study hypothesized that alginate intake by infected patients could reduce body inflammation, enhance their immune system, and increase antioxidant activity. Moreover, they also suggested/proposed that regular daily intake of alginate by healthy individuals could boost their immune system and may provide additional protection against SARS-CoV-2 infection. The statements presented above were based on an analysis of T lymphocytes. After investigating several COVID-19 patients, it was proven that a reduction in T-cell lymphocytes had a direct consequence on immune deficiency and inflammation caused by a viral infection. Studies on sulfated alginate derivatives have shown that they have antioxidant, anti-inflammatory, and immune effects on human macrophages. Moreover, alginate is the only natural marine polysaccharide that possesses antioxidant activity, immune-activating properties, biocompatibility, and nontoxicity [102].

Depending on the stage of the disease, diverse types of drugs can be administrated. Usually, drugs for supportive treatment, such as antiviral drugs, anti-inflammatory drugs, and monoclonal antibodies, are given. Today’s medical market includes drugs such as ribavirin [100], lopinavir/ritonavir [101], remdesivir [103], darunavir [104], favipiravir [105], sofosbuvir [106], dexamethasone [107], and chloroquine [103]. The mechanism of action of these drugs ranges from targeting viral proteins to targeting cellular proteins by increasing the immune system response to the viral infection [108]. Unfortunately, most antiviral drugs are not appropriate for oral or intravenous administration due to their low bioavailability and limited circulation. Thus, biomaterials may address these difficulties by serving as new carriers in antiviral treatment in the form of micro- or nanoparticles or capsules [109].

The capability of biomaterial-based drug delivery systems to target the lungs is very promising for treating COVID-19. The literature provides abundant information on biomaterial-based drug systems that are applied in the treatment of numerous disorders, such as lung cancer and influenza [110,111]. Natural biodegradable polymers such as cyclodextrin, albumin, alginate, and chitosan and synthetic polymers including polylactide (PLA), polyglycolide (PLGA), polyacrylates, and polyanhydrides are promising drugs for developing pulmonary therapeutic delivery systems for COVID-19 [112]. Based on the presented aspects, alginate-based biomaterials could be considered an important milestone/breakthrough in developing novel drugs/devices/platforms as a possible treatment against the coronavirus. 

The current study provides detailed information about the involvement of the cardiovascular system in coronavirus disease complications. The greatest priorities are curing infected patients who have developed cardiovascular complications, including myocardial injury, heart failure, or arrhythmia; uncovering the mechanism by which COVID-19 affects CVDs; and developing new treatment strategies [113].

Though the cause of their connection is not fully understood yet, it has been discovered that reactive oxygen species (ROS) play an important part in the evolution of CVDs. ROS are chemically reactive molecules containing oxygen. Disequilibrium between antioxidant defence mechanisms and ROS production induces oxidative stress, which leads to tissue injury and respiratory deficiency because hemoglobin is partially blocked by these species, and consequently, the oxygen demand is not met by the available oxygen carried by the blood [114,115].

Future perspectives about coronavirus outcomes are not optimistic. Besides the alarming prediction by epidemiologists that 40–70% of the population will be infected and the overwhelming demands on healthcare staff, there is a crucial need to provide new resources and develop comprehensive guidelines to overcome this pandemic [113,116]. Even if healthcare systems are overwhelmed, the best care must be provided equally to all patients regardless of their COVID-19 status, particularly to populations with pre-existing CVDs who appear to be more likely to be severely affected by the coronavirus. To reopen and normalize society, we must prepare for the post-COVID-19 period, with a special focus on prioritizing care for CVD and COVID-19 patients, who need to be closely monitored. As mentioned before, researchers must address the existing policies and strategies as a baseline to prepare for a similar future crisis [117].

The COVID-19 pandemic has had a crucial impact on a global scale. It has become evident that patients with cardiovascular comorbidities are most likely to be affected by severe COVID-19. Currently, complications in COVID-19 patients must be closely monitored to ensure that outcomes do not worsen. Considering all these facts, further follow-up must be considered, and researchers around the world need to come together to determine the cause of this pandemic [118,119,120].

## 7. Conclusions

This manuscript highlights the source of origin, unique properties, and biomedical applications of alginate. Alginate has received the most attention for drug delivery systems, but the most important prospect remains developing new devices for repairing damaged heart tissues affected by the coronavirus. Alginate has numerous advantageous properties, such as biocompatibility, biodegradability, nontoxicity, and easy gelation, which are important features for its use in numerous applications in diverse fields, such as the food and beverage industry but also in high-tech fields such as biotechnology or bio (/nano/) medicine. This review also presents a summary of diverse examples of alginate-based materials used in developing innovative devices for CVDs. Among the biomedical applications of alginate-based nanocomposites presented in this manuscript, some suitable examples that could yield significant benefits in the field of tissue engineering are worth mentioning. The excellent antimicrobial and anti-inflammatory activity of alginate indicate its potential use in biomedical applications. Alginate has made important contributions to other medical applications, including the pharmaceutical industry and dental practice, where it is used in capsules and tablets for targeted administration and bonding platforms for dental applications. The synergism between alginate’s properties and chemical modification for functionalization is crucial in applications such as tissue engineering. It was found that injecting 3D-printed biodegradable alginate hydrogel scaffolds have potential in the reconstruction of bones, cartilage, blood vessels, organs, and other tissues. In addition to the examples above, injectable alginate implants have proven to be a promising prospect for use in myocardial repair and regeneration. Moreover, introducing reduced graphene oxide to alginate has led to considerable improvements in its biocompatibility, and electric stimulation and electric signal transmission of alginate are important in developing new platforms for cardiac repair. It is worth mentioning that alginate hydrogels would have great potential as treatment platforms in the field of cardiovascular disease, and alginate’s applicability may be broadened and possibly contribute to overcoming COVID-19 complications among patients with heart failure. Based on the research progress in developing biomaterial-based antiviral platforms, alginate-based materials can be considered an important breakthrough as possible platforms for the treatment against COVID-19.

## Figures and Tables

**Table 1:** Potential applications of alginate-based materials.

No.	Alginate-Based Materials	Potential Applications	Findings	References
1	Alginate	Pharmaceutical	The development of novel polymers is useful for medical drugs.	[5,29,66,67]
2	Alginate gel	Cardiovascular	Alginate can be applied in the design of solutions for the treatment of cardiovascular diseases and the creation of heart valves, blood vessels, and drug and stem cell delivery systems.	[6]
3	Alginate hydrogels	Wound healing dressings	Composite hydrogels were developed as a wound dressing for healing wounds.	[7,8]
4	Alginate hydrogels	Cardiac tissue engineering	Alginate hydrogels were used for the fabrication of heart valve tissue engineering.	[9,66]
5	Alginate hydrogels	Tissue engineering	Injectable alginate hydrogels were used for cell delivery in tissue engineering.	[10]
6	Alginate–reduced graphene oxide	Cardiac repair	The prepared alginate–reduced GO electroactive hydrogel was used as a platform for stem cell therapy.	[11]
7	Alginate matrices	Drug delivery	Alginate polymeric matrices were developed for drug delivery applications.	[12]
8	Alginate polymers	Biomedical	Pseudomonads cultured from several cases of cystic fibrosis were described.	[13,18]
9	Alginate films	Food	Biodegradable polysaccharides have the potential for food packaging applications.	[14,24]
10	Alginate matrices	Regenerative	Alginate formulations of porous scaffolding matrices of cell culture were developed for regenerative medicine applications.	[30,49]
11	Alginate matrices	Tissue engineering	Alginate polymers can serve as matrix delivery vehicles for gene carriers and tissue engineering scaffolds.	[31]
12	Alginate hydrogel	Drug delivery	Alginate-based polymers can function as oral delivery matrices for proteins.	[31,32]
13	Lysozyme chitosan–alginate microspheres	Pharmaceutical	Lysozyme-containing chitosan-coated alginate microspheres systems can be applied for oral immunization with microencapsulated antigens.	[39]
14	Alginate scaffolds	Myocardial tissue engineering	Alginate scaffolds were designed for myocardial tissue engineering.	[44]
15	Alginate hydrogels as scaffolds	Tissue engineering	Alginate gels and gel/cell systems were formulated for tissue engineering applications.	[45]
16	Alginate hydrogels as drug delivery carriers	Biomedical	Alginate hydrogels were studied as drug and cell carriers and as tissue engineering matrices.	[46]
17	Injectable calcium phosphate–alginate hydrogel–umbilical cord mesenchymal stem cell paste	Bone tissue engineering	The injectable stem cell construct is based on calcium phosphate–alginate hydrogel for bone tissue engineering.	[47]
18	Alginate–ceramic composite materials	Bone tissue engineering	Alginate encapsulated murine-derived adipose-tissue stromal cells may be suitable as injectable bone graft substitutes.	[50]
19	Core–shell fibrous collagen–alginate hydrogel	Bone tissue engineering	The newly designed core–shell collagen–alginate fibrous carrier enables the encapsulation of tissue cells and their delivery into damaged targeted tissue to promote bone tissue engineering.	[54]
20	Gallium 3D alginate-coated bioglass scaffolds	Bone tissue engineering	Novel gallium-loaded 45S5 bioglass-based scaffolds coated with alginate are a candidate for bone tissue engineering.	[56]
21	Polycaprolactone–alginate–chondrocyte scaffolds	Cartilage tissue engineering	Polycaprolactone–alginate–chondrocyte scaffold is an innovative cell-printed scaffold for cartilage regeneration fabricated by advanced bioprinting technology.	[58]
22	Nanocellulose–alginate	Cartilage tissue engineering	Nanocellulose–alginate bioink is a suitable hydrogel for 3D bioprinting of living tissues and organs.	[59]
23	Alginate scaffolds	Liver tissue engineering	Alginate scaffolds provide a favorable microenvironment for new liver tissue creation and regeneration.	[62]
24	Alginate–gelatin hydrogels	Muscle tissue engineering	Oxidized alginate–gelatin hydrogels could be a suitable candidate for muscle tissue engineering.	[63]
25	Fibrinogen-modified sodium alginate scaffolds	Skin tissue engineering	Thrombin-modified alginate sponges can be successfully used as a grafting material to promote skin healing and regeneration.	[64]
26	Alginate hydrogels	Adipose tissue engineering	Alginate hydrogel has promising applications in soft tissue engineering.	[65]
27	Alginate beads immobilized on a polyurethane matrix	Biomedical	Alginate-based polyurethane can modernize the food and biomedical industries.	[67]
28	Graphene mesh loaded with netrin-1 supported by alginate	Peripheral nerve regeneration	The hydrogel nerve scaffold can significantly promote the regeneration of peripheral nerves and restoration of denervated muscles.	[68]
29	Polyacrylamide/graphene oxide/gelatin/sodium alginate composite hydrogel	Peripheral nerve regeneration	The design and development of hydrogel scaffolds provide an important experimental basis for nerve tissue engineering applications.	[69]
30	Alginate hydrogels in regenerative and therapeutic medicine	Biomedical	Alginate hydrogels are solutions for creating heart valves, blood vessels, and drug/stem cell delivery vehicles.	[6]
31	Montmorillonite–alginate nanocomposite as drug delivery systems in chemotherapy	Biomedical	Nanocomposite beads based on montmorillonite–alginate may be a promising drug delivery system.	[70]
32	Alginate-based matrix	Pharmaceutical	Alginate-based matrix tablets were used in modified drug delivery formulations using metronidazole as a model drug.	[71]
33	Doxorubicin-loaded glycyrrhetinic acid-modified alginate nanoparticles	Biomedical (clinical)	Heart and liver cells surrounding the tumor were not affected by drug intake.	[72]
34	Calcium alginate	Wound healing	Calcium alginate is more appropriate for topical treatment of diabetic foot lesions.	[73,74]

## Data Availability

Data sharing not applicable.
